# Update in TIGIT Immune-Checkpoint Role in Cancer

**DOI:** 10.3389/fonc.2022.871085

**Published:** 2022-05-17

**Authors:** Tiziana Annese, Roberto Tamma, Domenico Ribatti

**Affiliations:** ^1^ Department of Medicine and Surgery, Libera Università del Mediterraneo (LUM) Giuseppe Degennaro University, Bari, Italy; ^2^ Department of Basic Medical Sciences, Neurosciences and Sensory Organs, Section of Human Anatomy and Histology, University of Bari Medical School, Bari, Italy

**Keywords:** cancer, immune-checkpoint, immune-therapy, TIGIT, tumor microenvironment

## Abstract

The in-depth characterization of cross-talk between tumor cells and T cells in solid and hematological malignancies will have to be considered to develop new therapeutical strategies concerning the reactivation and maintenance of patient-specific antitumor responses within the patient tumor microenvironment. Activation of immune cells depends on a delicate balance between activating and inhibitory signals mediated by different receptors. T cell immunoreceptor with immunoglobulin and ITIM domain (TIGIT) is an inhibitory receptor expressed by regulatory T cells (Tregs), activated T cells, and natural killer (NK) cells. TIGIT pathway regulates T cell-mediated tumor recognition *in vivo* and *in vitro* and represents an exciting target for checkpoint blockade immunotherapy. TIGIT blockade as monotherapy or in combination with other inhibitor receptors or drugs is emerging in clinical trials in patients with cancer. The purpose of this review is to update the role of TIGIT in cancer progression, looking at TIGIT pathways that are often upregulated in immune cells and at possible therapeutic strategies to avoid tumor aggressiveness, drug resistance, and treatment side effects. However, in the first part, we overviewed the role of immune checkpoints in immunoediting, the TIGIT structure and ligands, and summarized the key immune cells that express TIGIT.

## Introduction

Solid and hematological malignancies are complex ecosystems that arise from malfunctioning complex cellular mechanisms controlled by genetic and epigenetic factors that coordinate the cross-talk between tumor cells and the tumor microenvironment (TME) components. Among the cellular components of the TME, T cells are the second most abundant cell type after tumor-associated macrophages (TAMs) (1).

**Figure 1:**
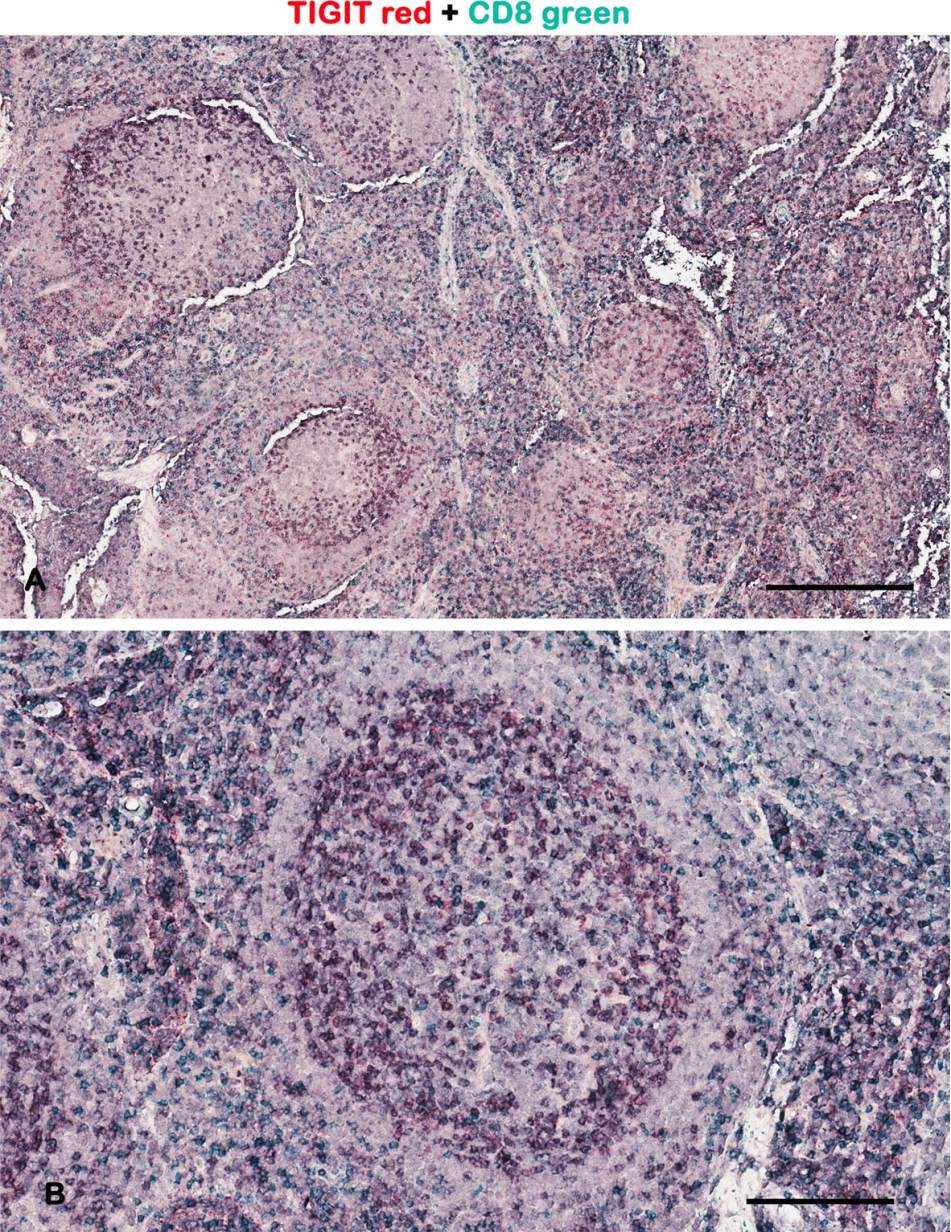
Representative brightfield images of double IHC for TIGIT and CD8 in a lymph node. Micrographs show TIGIT staining in red, CD8 staining in green, and the colocalized TIGIT^+^CD8^+^ signals in purple. As demonstrated by other authors, the TIGIT^+^ T cells are preferentially at the periphery of the germinal center (2). Scale bar: **(A)** 500 μm; **(B)** 165 μm.

Following the development phase in the thymus, the diverse naïve T cells migrate to the secondary lymphoid organs, where they remain dormant until they are activated by recognition of the antigen-human leukocyte antigen (HLA) complex presented by the APC to their TCR (**Figure 1**). In addition to antigen recognition by TCR, naïve T cell activation is regulated by second signals known as co-stimulatory pathways, such as the well-noted CD28–CD80/CD86 and CTLA4–CD80/CD86 (3, 4). These co-stimulatory pathways have a lot of receptor/ligand pairs, also called immune checkpoints, which lead to positive signaling events, while other pathways send out negative signals (5).

CD28 is constitutively expressed on naïve CD4^+^ and CD8^+^ T cells, while CD80 and CD86 are inducible on APCs. CD28–CD80/CD86 pathway activates T cell responses. Other stimulatory immune checkpoints are members of the tumor necrosis factor (TNF) receptor superfamily (CD27, CD40, OX40, GITR, and CD137) or the B7–CD28 superfamily (ICOS) (6).

On the contrary, cytotoxic T-lymphocyte-associated protein 4 (CTLA4) is a negative regulatory inducible receptor for CD80/CD86 and has inhibitory effects on T cell responses, leading to T cell attenuation and tumor cell immune evasion (7). A considerable number of inhibitory immunoreceptors have been identified and studied in tumors, including but not limited to adenosine A2A receptor (A2AR), B7-H3, B7-H4, programmed death (PD-1), CTLA4, T cell immunoglobulin domain and mucin domain 3 (TIM3), T-cell immunoreceptor with immunoglobulin and ITIM domain (TIGIT), and B and T lymphocyte attenuator (BTLA) (6, 8).

Interestingly, the immune system can constrain and promote tumor development and progression (5, 9, 10). Alterations in immune checkpoint pathways result in an imbalance of positive and negative co-stimulatory signals, which increases the risk of tumorigenesis and its progression. These signals are also involved in patients’ resistance to immunotherapies (9, 11).

Immune checkpoint inhibitors were developed to block checkpoints by making T cells free to attack cancer cells. These therapies are also referred to as checkpoint blockade therapies and are an emerging and attractive field to treat many cancers, but they do not work for all patients and can cause serious side effects (12). The failure of classical antitumor therapies could be attributed to the fact that most drugs currently in use primarily target tumor cells and not also TME cells. These cells are different cell types, including endothelial cells, stromal cells, and immune cells. Understanding the *in situ* cross-talk of heterogeneous tumor cells with various tumor-associated immune cells, such as T cells, will provide critical information for improving anticancer therapies.

The immune checkpoint inhibitors are most efficacious in patients with a TME enriched in tumor-infiltrating lymphocytes (TILs) (13). TILs are deputed to tumor immunoediting [“a dynamic process wherein immunity functions not only as an extrinsic tumor suppressor but also to shape tumor immunogenicity” (14)]. Immunoediting shapes tumor fate in three steps: elimination, equilibrium, and escape. The elimination step is the immunosurveillance step, in which a competent immune system (innate and adaptive immunity) recognizes and destroys transformed cells expressing highly immunogenic antigens long before they become clinically relevant (15).

If some cancer cells evade the elimination step, they will enter the equilibrium step, in which survived tumor cells and immune cells mutually edit each other. During this adaptation time, tumor cells undergo a complex process of natural selection [similar to that described by Darwin (16)] that presses on tumor cells with traits that are better suited to the environment than others.

These natural evolution-selected tumor cell variants develop resistance to elimination and put them in the escape step (17). A progressive establishment of an immunosuppressive TME characterizes the escape step (11, 18). This is the final step when aggressive-selected tumor clones develop diverse ways to escape the immune system that mimic peripheral tolerance (8, 19, 20): prevent the response of effector T cells, TAMs, natural killer (NK) cells, and tumor-associated neutrophils (TANs) (21); down-regulate their HLA (22); induce antigen presentation defects; eliminate neoantigens; inhibit immune cell chemoattraction to the tumor site; secrete or promote the secretion of immunosuppressive cytokines (23); modulate the recruitment and expansion of immunosuppressive cells, such as regulatory T cells (Tregs); orchestrate immune cell metabolism (24); and activate immune checkpoint pathways to inhibit the emerging antitumor immune response (25).

T cells immunoediting also occurs during tumorigenesis (26, 27). At first, in order to attack and eliminate tumor cells, APCs, *via* CD28-CD80/CD86 pathway, activate T cells, but at the same time regulate pro-inflammatory mechanisms, activating inhibitory pathways by immune checkpoints (28). Among immune checkpoint inhibitors, immediately after TCR engagement, CTLA4 is upregulated and competes with CD28 to bind to CD80/CD86 on APCs, limiting autoreactive T cells, decreasing T cell priming and proliferation, inducing immune tolerance, and preventing autoimmunity (29, 30).

In the immune response, PD-1 is also expressed on activated T cells, but it acts later and interferes with T cells that have already been activated (31). When the stimulating antigen is removed, PD-1 expression on responding T cells decreases, whereas it remains increased in the opposite scenario. Like CTLA4, the PD-1–PD-L1/PD-L2 pathway recruits phosphatases to block the stimulatory signals sent by TCR and CD28–CD80/CD86, resulting in decreased T cell activation, survival, cytokine generation, and metabolism (31). Overexpression of PD-1 on tumor cells or by the cellular component of the TME with its downstream pathway is a systematic strategy used by malignancies to increase exhausted T cells and to evade immunosurveillance. The fact that PD-1 overexpression happens later means that it will only be overexpressed and activated once an inflammatory process has begun (32).

Immune checkpoint activation and an immune infiltrate enriched in Tregs were identified as the primary tumor escape mechanisms in a mouse model of hypermutated and microsatellite-instable colorectal cancer (33). According to the same study, the expansion of the TCR following PD-1 blockade potentiates immunoediting (33).

In a subtype of advanced untreated primary colorectal cancer, immune checkpoints expression has been related to immune evasion *via* neo-antigen-related mechanisms (34). This subtype was called the “stealth subtype,” and immune evasion and poor prognosis have been associated with less clonal highly expressed neoantigens (HiNeo), high chromosomal instability, high immune checkpoint expression (PD-1, PD-L1, PD-L2), low neoantigen presentation (reduced HLAII), downregulation of functional CD8^+^ T cells, and a microenvironment poor in TAMs and B cells (34, 35).

After T cell activation, also TIGIT expression increases on T cells, where it competes with CD226 (DNAM-1) for binding to their shared ligands CD112 and CD155 (36). TIGIT expression is late in the cancer-immunity cycle. It is highly expressed on specialized CD4^+^ subsets, such as Treg and T_FH_, and lowly expressed on CD4^+^ and CD8^+^ exhausted T cells (37). Moreover, TIGIT^+^CD4^+^ T cells and TIGIT^+^CD8^+^ T cells displayed a memory phenotype (37).

The timing of immune checkpoint activation is currently under investigation because there is a debate about the reactivation of primed T cells and/or novel T cells. The former depends on memory T cells and presumes the existence of pre-existing cancer-specific T cells that recognize tumor-specific antigens. The second depends on novel T cells against neo-antigens and therefore assumes that T cells are primed and recruited to tumors after the initiation of therapy (38–40). This topic is interesting in patient stratification for immune checkpoint blockades therapy since the success of these therapies relies on antigen processing and presentation (41–43).

In this context, the TIGIT immune checkpoint is emerging as a promising target for anticancer therapy alone or combined with other immune blockade therapies (44). The purpose of this review is to update the role of TIGIT in cancer progression, looking at last year’s studies about its pathways that are often upregulated in immune cells and possible therapeutic strategies to avoid tumor aggressiveness, drug resistance, and treatment side effects. However, in the first part, we overviewed the TIGIT structure and ligands, and summarized the key immune cells that express TIGIT.

## Overview Of TIGIT Structure and Ligands

TIGIT is also known as V-set and immunoglobulin domain-containing protein 9 (VSIG9) or V-set and transmembrane domain-containing protein 3 (VSTM3). Based on UniProt data resources, two alternatively spliced isoforms have been reported in humans.

It has an extracellular Ig-like V-type domain, a type I transmembrane domain, and a cytoplasmic domain with the immunoreceptor tyrosine-based inhibitor motif (ITIM) (45, 46) and the immunoglobulin tyrosine tail (ITT)-like motif (45, 47). ITIM modulates cellular responses by binding the SH2 domain of several SH2-containing tyrosine phosphatases, SHP1 (48) and SHP2, when phosphorylated (49).

After the extracellular ligand binding, the ITT-like domain is phosphorylated at Tyr225, binds the two cytosolic adaptor proteins Grb2 and β-arrestin2, and recruits the SH2-containing inositol phosphatase-1 (SHIP-1) that inhibits PI3K/MAPK signaling (via Grb2) (50) to reduce the NK cell effector functions (50), and TRAF6/NF-κB signaling (via β-arrestin2) to inhibit IFN-γ production (48).

Different studies show that phosphorylation of the tyrosine residue in either ITIM- (Y231) or ITT-like (Y225) motif is essential for signal transduction and the inhibitory function of TIGIT in humans (48, 50). When both tyrosine residues are mutated, the inhibitory activity of human TIGIT is completely lost (51).

TIGIT binds to nectin and nectin-like (NECL) adhesion molecules, including NECTIN-2 (CD112) (52, 53), NECTIN-3 (CD113) (45, 51), and NECL-5 (CD155) (54) to mediate cell adhesion and signaling.

TIGIT binds NECL in cis-trans, forming a receptor clustering. For instance, two TIGIT–CD155 dimers assemble into a heterotetramer with a core TIGIT–TIGIT cis-homodimer in which each TIGIT molecule binds one CD155 molecule (47).

CD112 is a cell adhesion protein involved in the modulation of T cell signaling. Two isoforms, delta and alpha, are annotated by alternative splicing. Depending on the receptor it binds to, CD112 can be either a co-stimulator or a co-inhibitor of T cell function: CD226 binding stimulates T cell proliferation and cytokine production (IL2, IL5, IL10, IL13, and IFN-γ) (55); PVRIG (also called CD112R) binding inhibits T cell proliferation (56). These interactions are competitive (57). CD112 binds with low affinity to TIGIT (46, 52). The TIGIT binds to CD112 destroys CD112–CD112 homodimer (52) and, as for TIGIT–CD155, homo- and heterodimers in the heterotetramer interact by a conserved “lock and key” binding (52). CD112 is highly expressed in bone marrow, kidney, pancreas, lung cells, and breast and ovarian cancer (58, 59).

CD113 is another cell adhesion protein that interacts with nectin and NECL molecules *via* heterophilic trans-interactions, such as CD112 at Sertoli-spermatid junctions (60). Through common signaling molecules such as SRC and RAP1, CD113 trans-interaction with CD155 activates CDC42 and RAC small G proteins (61). CD113 also establishes cell-cell junctions, such as adherens junctions and synapses (62, 63). It inhibits cell movement and proliferation by inducing endocytosis-mediated downregulation of CD155 on the cell surface (64). CD113 contributes to the morphology of the ciliary body (65). CD113 is highly expressed in the testis, placenta, kidney, liver, and lung (66). CD113, like CD112, has a low affinity for TIGIT, and their interaction prevents the self-destruction of normal cells by NK cells (46, 53).

CD155, the primary ligand for TIGIT, is also known as the Poliovirus receptor (PVR). CD155 has a “lock-and-key” motif that is essential for TIGIT binding and is highly conserved across PVR family members (47). CD155 is a glycoprotein with three extracellular immunoglobulin domains, transmembrane, and intracellular domains (67). Two splice forms, lacking the transmembrane region, have also been described as soluble or secreted isoforms that seem to compete with the membrane-anchored ones (68, 69). CD155 is highly expressed on CD11c^+^ human dendritic cells (DCs) (70, 71), macrophages (72, 73), T (74) and B cells (75), epithelial cells (74, 76), kidneys (76), nervous system (77), intestine (78, 79), and tumor cells (80, 81). *In vivo*, the CD155–TIGIT pathway suppresses immunological responses increasing IL-10 anti-inflammatory cytokine (82, 83) and decreasing IL-12 pro-inflammatory cytokine released by DCs (46, 84). This induces a tolerogenic phenotype in T cells (85). A more detailed description of the CD155–TIGIT pathway in cancer is present in the next section.

A new NECL that exclusively binds TIGIT was recently identified, NECTIN-4 (86). TIGIT binds NECTIN-4 with high affinity, comparable to CD155 (86). NECTIN-4 is involved in cell adhesion through trans-homophilic and -heterophilic interactions, including specific interactions with NECTIN-1 (CD111) (87), does not interact with CD226, CD96, or CD112 (86), and is overexpressed in several tumors of the breast (88, 89), bladder (90), lung (91, 92), and pancreas (93, 94).

## TIGIT Pathways and Immune Cells Involved

TIGIT is expressed by a variety of immune cells. Its expression and related pathways have been discussed in this section.

**Figure 2:**
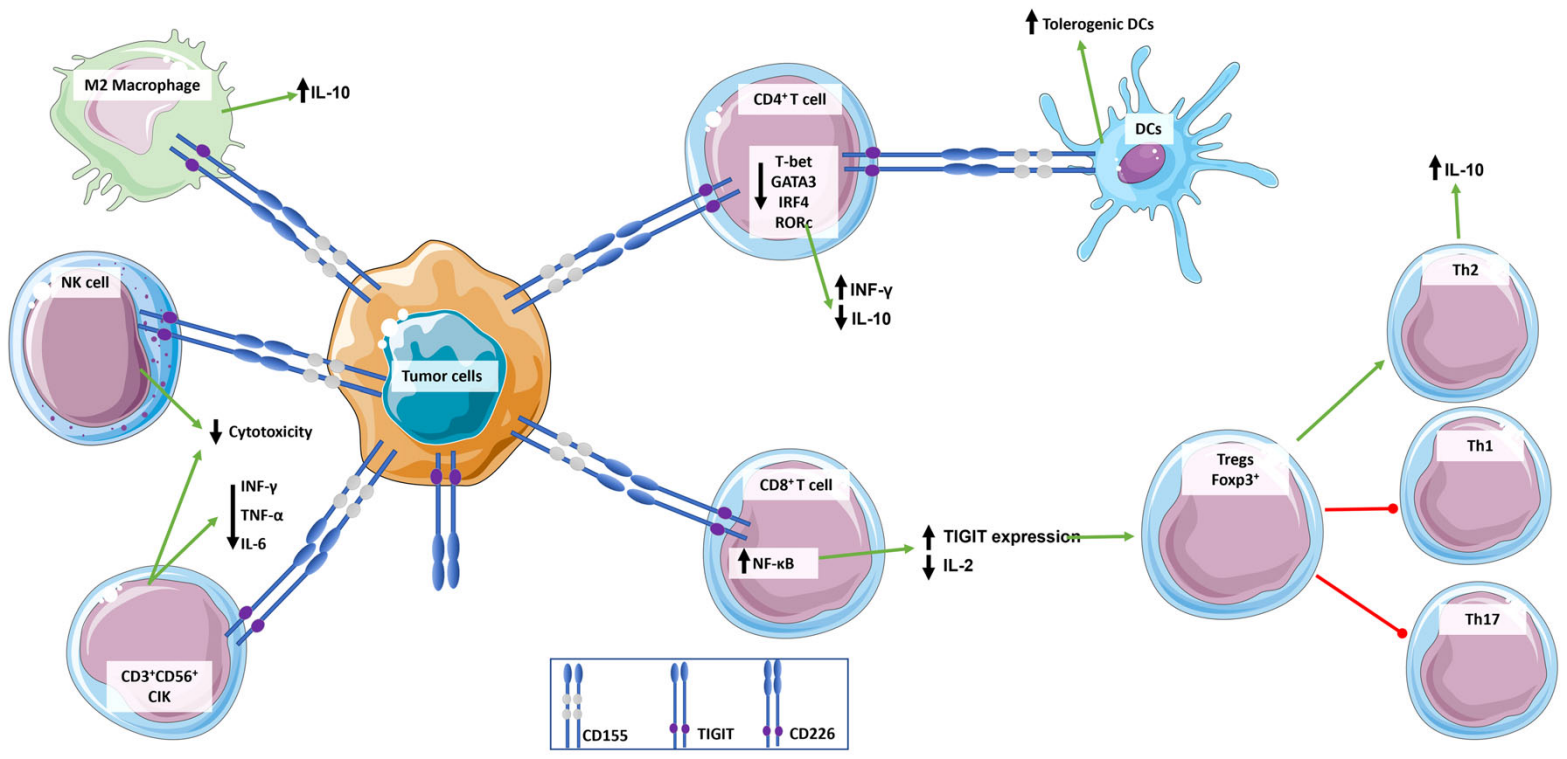
Role of TIGIT in the regulation of immune response. TIGIT transmits inhibitory signals *via* ITIM and immunoglobulin tyrosine tail (ITT)-like motifs in its cytoplasmic domain when it is engaged. TIGIT has multiple ligands, but it binds with greater affinity to CD155, which is widely expressed by immune cells and tumor cells. CD155 expressing tumor cells bind to TIGIT expressed by immune cells inducing an immunosuppressive and tolerogenic microenvironment: CD4^+^ T cells induce a tolerogenic phenotype in DCs, release the anti-inflammatory cytokine IL-10, and down-regulate INF-γ; CD8^+^ T cells up-express TIGIT and down-regulate the release of pro-inflammatory cytokine IL-2, which in turn promotes a T cell immunosuppressive phenotype characterized by increase in Foxp3^+^ Tregs and Th2 compared to pro-inflammatory Th1 and Th17; NKs cytotoxicity is suppressed; and macrophages switch to an M2 anti-inflammatory phenotype. This simplistic view does not integrate signals from the CD226/CD155 pathway.

In simple terms, TIGIT activation creates a tolerogenic microenvironment in both cell-intrinsic and cell-extrinsic ways (resumed in **Figure 2** and discussed in the following). This means that TIGIT competes directly with CD226 for binding to CD155, CD112, or CD113 ligands in the former way, or that it is involved in events that indirectly induce immunosuppressive effects, such as TIGIT’s action on innate immunity cells in the second way.

CD226 is a member of the immunoglobulin superfamily and consists of an extracellular region with two IgV-like domains, a transmembrane region, a cytoplasmic region with ITT, and four putative tyrosine residues and one serine residue that are phosphorylated (95). It is mainly expressed on myeloid and lymphoid cells (96), through which promotes intercellular adhesion, lymphocyte communication, and lymphokine production, as well as enhances cellular cytotoxicity mechanisms (96).

TIGIT–CD155 in CD4^+^ T cells induces immunosuppression inhibiting T cell proliferation directly by inducing the down-expression of T-bet, GATA3, IFN regulatory factor 4 (IRF4), and retinoic acid-related orphan receptor c (RORc), which reduce the level of pro-inflammatory IFN-γ while increasing the level of anti-inflammatory IL-10 (97).

TIGIT–CD155 in NK cells reduces their cytotoxicity (51, 53), resulting in impaired granule polarization and IFN-γ production (50, 98). On the contrary, TIGIT blockade restored potent effector NK cells through CD226–LFA-1 signaling that increases adhesion to target cells, induces IFN-γ production by naïve CD4^+^ T cells, and enhances the cytotoxic function of NK cells (99, 100).

TIGIT–CD155 signaling was also observed in cytokine-induced killer (CIK) cells expressing CD3 and CD56 molecules (101, 102). As observed indirectly by Zhang et al., who analyzed the literature concerning the clinical trial ongoing on renal cell carcinoma patients enrolled in integrated CIK cell immunotherapy, the TIGIT blocked enhanced CIK proliferation and the release of pro-inflammatory cytokines, such as IFN-γ, IL-6, and TNF-α (102).

TIGIT–CD155 in CD8^+^ T cells induces immunosuppression *via* the NF-κB signaling pathway, promoting a tolerant state that is passed down across T cell generation. In this process, CD155^+^ naïve T cells trans-interact with TIGIT^+^ preceding tolerant T cells resulting in increased TIGIT expression and IL-2 suppression *via* Blimp1 increment (54, 103).

TIGIT–CD155 signaling was also observed in activated Foxp3^+^ Tregs, which suppress pro-inflammatory Th1 and Th17 but not Th2 cells *via* Akt repression and FoxO1 phosphorylation, IL-10 and fibrinogen-like protein 2 overexpression (104, 105). According to this shift in immunity from Th1 and Th17 to Th2 immunity and IL-10 release, CD226 is expressed on Th1 and Th17, but not on Th2 cells, and in the former, CD226–CD155 promotes IFN-γ and IL-17 production (106, 107).

Concerning TIGIT-mediated tolerogenic microenvironment by cell-extrinsic ways, it was observed that TIGIT suppresses T cell function by enhancing the immunosuppressive function of DCs and macrophages that express TIGIT ligands such as CD155 (46, 97, 108, 109).

TIGIT^+^CD4^+^ T cells exerted immunosuppressive effects indirectly by modulating the monocyte‐derived DCs cytokine production (97). TIGIT of CD4^+^ T cells interacts with CD155^+^ of DCs, modulating the Erk signaling pathway and increasing IL-10 production while decreasing IL-12p40 production and promoting tolerogenic DCs that suppress T cell responses (46, 97).

TIGIT was found to play a role in macrophages in an *in vitro* pig-to-human xenograft model (84). In this model, TIGIT is expressed by M2 macrophages but not by M1 macrophages or endothelial cells. At the same time, CD155 is expressed by both M1 and M2 macrophages. Here, the immunosuppressive effects of TIGIT are explained by reduced expression of pro-inflammatory cytokines, such as TNFα, IL-1β, and IL-12 in M1 *via* SHP-1 phosphorylation. In BALB/c mice, TIGIT immunomodulates CD155^+^ pro-inflammatory M1 into IL-10-secreting anti-inflammatory M2 (85).

All of this demonstrates the intricacy of the several targets and pathways that ani-TIGIT immunotherapies must consider.

## TIGIT In Cancer Progression

Immune dysregulation may play a role in cancer progression (110). TIGIT overexpression has been found in the cellular microenvironment of several tumors, including lung (111), kidney (112), liver (113), glioma (114, 115), melanoma (116), colorectal carcinomas (117), gastric cancer (118), and neuroblastomas (119). TIGIT expression was found to be strongly associated with poor prognosis in colorectal cancer and positively correlated with pathological stages in renal clear cell carcinoma (120), kidney renal papillary cell carcinoma, and uveal melanoma (121, 122).

**Figure 3:**
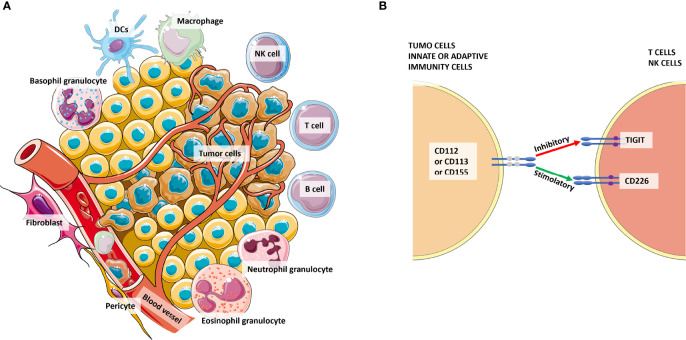
The complexity of the tumor microenvironment and focus on TIGIT^+^ cells. Panel **(A)** shows the major cellular components of the microenvironment that cross-talk with tumor cells. Panel **(B)** shows the competition among CD226 and TIGIT to bind their ligands CD112 or CD113 or CD155 expressed by tumor cells or antigen-presenting cells (APCs) from innate or adaptive immunity. Especially for CD155, the affinity for TIGIT is higher than its affinity for CD226. Thus, the signaling of the CD155-TIGIT synapse (red arrow) induces immunosuppression rather than effector cell activation and/or cytotoxicity.

As explained in the introduction paragraph, immune cells interact with other microenvironment cells and the tumor cells in a cross-talk that determines the cancer features and heterogenicity (123, 124). Chronic antigen exposure, which characterizes the first part of tumorigenesis when tumor cells become detectable, stresses T cells, causing them to lose their effector function, become exhausted, and upregulate several immune inhibitor receptors (IRs) such as TIGIT (125, 126) (**Figure 3**). In various cancers, according to computational analyses, the TIGIT expression profile was related to the immune infiltration level, coupled with the expression of other IRs, including LAG3, CTLA4, PD-1, PD-L1, PD-L2, and it is related to tumor mutation burden (TMB), microsatellite instability (MSI), mismatch repair (MMR), and DNA methyltransferases (DNMTs) gene alterations in different tumors (122). Gene set enrichment analysis (GSEA) demonstrated a negative association among high TIGIT expression and cytokine-cytokine receptor interaction, chemokine signaling pathway, NK-mediated cytotoxicity, allograft rejection, INF-γ response, and IL6/JAK/STAT3 signaling (122). On the contrary, a low TIGIT expression was associated with oxidative phosphorylation and propanoate metabolism (122).

Blocking the co-expression of IRs appears to be an excellent arm of immunotherapy. TIGIT co-expression with other IRs has been widely examined on CD8^+^ TILs and circulating T cells (116, 127). Li et al. demonstrated that distinct IRs are co-expressed on CD8^+^ TILs in T cell exhaustion of primary cancer treatment-naïve patients comprising breast, kidney, lung, liver, cervical, esophageal, gastric, and colorectal cancer (128). Almost 50% of CD8^+^ TILs were found PD-1^+^TIGIT^+^, indicating that TIGIT is preferentially co-expressed with PD-1 (128). Furthermore, in the same study for cervical cancer was observed that the advanced T cell differentiation (CD27^–^CCR7^–^CD45RA^–^) of PD-1^+^TIGIT^+^2B4^+^TIM3^+^KLRG-1^–^CTLA4^–^ CD8^+^ TILs was associated with 60% of poorly differentiated cervical cancer (128). TIGIT mono-expression was also highly present in both TILs and circulating T cells, and this is probably the cause of the side effects after systemic treatment with TIGIT blockade (128).

TIGIT and PD-1 high co-expression was observed in PBLs (peripheral blood lymphocytes), MALs (malignant ascites lymphocytes), and TILs with increased frequency in tumor proximity in matched samples of patients at first diagnosis of ovarian cancer not treated (129). Moreover, the authors also observed TIGIT and TIM3 co-expression in PBLs, MALs, and TILs but with a decreased frequency in tumor proximity (129).

Multiple IRs expression, such as PD-1, PD-L1, TIGIT, and CTLA4, was reported in detail in TILs and circulating T cells in primary breast cancer and colorectal cancer in which immune checkpoint expression was correlated with promoter demethylation and post-translational histone modifications (130–134). For instance, TIGIT in colorectal cancer and TIGIT plus PD-L1 in colorectal cancer and breast cancer were found hypomethylated at the gene promoter level (134).

In esophageal squamous cell carcinoma, the analysis of the RNA-seq dataset from The Cancer Genome Atlas (TCGA) database and by multiplex-immunohistochemistry reactions on patient’s biopsies revealed a high expression of PD-L1 with TIM3 or TIGIT (135). This high IRs co-expression was positively correlated to a greater extent with CD8^+^ TILs and to a lesser extent with CD4^+^ TILs and was associated with poor overall survival (OS), TNM III/IV stage, and short restricted mean survival time (RMST) (135).

A TIGIT role in T cell exhaustion was also reported in chronic lymphocytic leukemia (CLL) (136). By flow cytometric and transcript expression analysis, Hajiasghar-Sharbaf et al. have observed a significantly high number of TIGIT^+^PD-1^+^CD8^+^ T cells (136), PD-1^+^TIM3^+^CD8 T cells (137), and PD-1^+^TIM3^+^CD4 T cells (138) in CLL patients compared with control, particularly in patients with advanced TNM stage.

In both myeloid leukemia and multiple myeloma, using flow cytometry, the bone marrow resident γδ T cells, a T cell subpopulation of non-MHC-restricted, have shown TIGIT, PD-1, TIM3, and the ectonucleoside triphosphate diphosphohydrolase-1 (CD39) co-expression at a high level compared to αβ T cell but similar to that expressed on CD8^+^ effector T cells (139). These markers were linked to signs of exhaustion, such as transcriptional reprogramming, decreased release of proinflammatory cytokines, decreased T cell proliferation, and lesser tumoricidal activity, and were associated with a lower OS for myeloid leukemia (139, 140).

In relapsed/refractory classic Hodgkin’s lymphoma, a TIGIT-mediated alternative system of immune escape was demonstrated to the classic PD-1/PD-L1 (141). TIGIT and PD-L1 were found to be mutually exclusively expressed and TIGIT^+^PD-1^+^CD3^+^CD4^+^T cells surrounding Hodgkin Reed-Sternberg (HRS) cells were associated with advanced TNM stages (141).

IRs blockades are mainly used for T cells, but also NK cells could be a valid target for immunotherapy (142, 143). The expression pattern of immune checkpoints on NK cells isolated from peripheral blood of patients affected by hepatitis B virus-related hepatocellular carcinoma (HBV-HCC) revealed a positive correlation among the co-expression of TIGIT and TIM3 in exhausted T cells, high rate of tumor progression, and poor clinical prognosis (144).

In melanoma patients, tumor-infiltrating NK cells were present at low frequencies in metastatic melanoma, had downregulated expression of both TIGIT and CD226, and *in vitro* experiments had shown their dysfunctional phenotype with higher lytic potential but lower lytic activity compared with TIGIT^−^ NK cells against CD155^+^ MHC class I–deficient melanoma cells (145). Interestingly, in the same study, TIGIT blockade as a single treatment failed to reverse NK cells dysfunction, while together with IL-15 had reversed CD155-mediated NKs exhaustion and had inhibited experimental melanoma metastasis *in vivo* (145).

Despite their inhibitory effects on T cells, PD-1 and TIGIT co-expression were described in activated T cells with a cytotoxic effector phenotype and the CXCR5 overexpression (146–148). In Merkel cell carcinoma patients, the PD-1^+^TIGIT^+^ CD8^+^ T cells circulating population was significantly associated with clinical benefit (146). Moreover, a positive trend, but not significant, was observed in melanoma patients (146). In both diseases, the monitoring of PD-1^+^TIGIT^+^ CD8^+^ T cells was proposed as a predictive biomarker of clinical efficacy for PD-1 blockade (146).

Though under-investigated, TIGIT is also expressed in CD4^+^ Tregs in association with an increased hypomethylation state (149, 150). In melanoma patients, increased TIGIT/CD226 ratio was observed in CD4^+^ Tregs compared with CD4^+^ effector T cells and was associated with highly suppressive TME and poor clinical outcomes (149). TIGIT hypomethylation was found dependent by Foxp3. It is a marker of CD4^+^ Tregs and works as a transcriptional activator by binding to demethylated sequences containing a Forkhead-binding motif, as observed in TIGIT, MIR21, FOXP3, CTLA4, and CD25 (128, 150). Altogether, these data demonstrated that epigenetic regulators, such as demethylation inhibitors, together with immune checkpoint inhibitors, should be considered in new combined therapeutical approaches, and that the promoter methylation pattern of immune checkpoints could be a valid prognostic biomarker.

Here, we discern last year’s update concerning TIGIT’s role in cancer based on PubMed search [for an update concerning hematological malignances, see the review (144)]. We have also looked at studies investigating the correlation of TIGIT expression with the clinicopathological characteristics of such a tumor (such as grade, stage, and metastasis) to improve clinical diagnosis, the amount of surgical resection, prognosis determination, and target therapy. Indeed, a 2021 meta-analysis of TIGIT expression in the tumor microenvironment of various solid tumors revealed that it has prognostic value because it is associated with risk factors for OS and progression-free survival (PFS) (142). In **Table 1**, all the clinical trials evaluating anti-TIGIT immunotherapeutics started in 2021 are collected, while an in-depth discussion on TIGIT in clinical development is elegantly presented by Rotte et al. (143). (Note that there are now “new” cancers as glioblastoma and melanoma in the clinical trials and not only the “usual” lung cancers. This will give important clinical data on TIGIT blockade on different tumors).

**Table 1:** Clinical trials evaluating anti-TIGIT immunotherapeutics started in 2021 (accessed on March 14, 2022).

NCT Number	Interventstions/Drug	Conditions	Status	Phases	Start Date
NCT05251948	Atezolizumab Capecitabine Oxaliplatin Tiragolumab	Gastric and gastroesophageal junction carcinoma	Recruiting	Phase 1 Phase 2	March 1, 2022
NCT05253105	TAB006 Toripalimab	Previously treated, advanced malignancies	Not yet recruiting	Phase 1	March 15, 2022
NCT05130177	Zimberelimab Domvanalimab	Melanoma	Not yet recruiting	Phase 2	March 2022
NCT05120375	BAT6021	Solid tumor	Not yet recruiting	Phase 1	Not avilable
NCT05102214	HLX301	Locally advanced or metastatic solid tumors Non-small cell lung cancer	Recruiting	Phase 2	January 2022
NCT05073484	BAT6021 BAT1308	Advanced solid tumor	Recruiting	Phase 1	October 29, 2021
NCT05060432	EOS-448 Anti-PD1 inupadenant	Advanced cancer Lung cancer Head and neck cancer Melanoma	Recruiting	Phase 1 Phase 2	September 6, 2021
NCT05061628	JS006 as Monotherapy JS006 in combination with Toripalimab	Advanced tumors	Recruiting	Phase 1	April 21, 2021
NCT05026606	Etigilimab Nivolumab	Recurrent fallopian tube clear cell adenocarcinoma Recurrent ovarian clear cell adenocarcinoma Recurrent platinum-resistant fallopian tube carcinoma Recurrent platinum-resistant ovarian carcinoma Recurrent platinum-resistant primary peritoneal carcinoma Recurrent primary peritoneal clear cell adenocarcinoma	Recruiting	Phase 2	October 1, 2021
NCT05023109	GP+PD-1+Tight	Biliary tract carcinoma	Not yet recruiting	Phase 2	September 1, 2021
NCT05019677	GP+PD-1+Tight	Intrahepatic cholangiocarcinoma	Not yet recruiting	Phase 2	September 1, 2021
NCT05014815	Ociperlimab Tislelizumab histology-based chemotherapy Placebo	Non-small cell lung cancer	Recruiting	Phase 2	November 16, 2021
NCT05009069	Radiotherapy Capecitabine Fluorouracil Atezolizumab Tiragolumab	Rectal neoplasms Rectal Cancer	Not yet recruiting	Phase 2	April 30, 2022
NCT04995523	AZD2936	Non-small cell lung carcinoma	Recruiting	Phase 1 Phase 2	September 14, 2021
NCT04952597	Ociperlimab Tislelizumab Concurrent Chemoradiotherapy	Limited stage small cell lung cancer	Recruiting	Phase 2	July 15, 2021
NCT04933227	Atezolizumab Tiragolumab Oxaliplatin Capecitabine	Stomach neoplasms Gastric cancer Gastroesophageal junction adenocarcinoma	Recruiting	Phase 2	August 6, 2021
NCT04866017	Tislelizumab Durvalumab Chemotherapy Ociperlimab	Non-small cell lung cancer	Recruiting	Phase 3	June 17, 2021
NCT04791839	Zimberelimab Domvanalimab Etrumadenant	Non-small cell lung cancer Non-small cell carcinoma Non-small cell lung cancer	Recruiting	Phase 2	August 4, 2021
NCT04761198	Etigilimab dosing Nivolumab	Solid tumor, adult Advanced solid tumor Metastatic solid tumor	Recruiting	Phase 1 Phase 2	March 23, 2021
NCT04746924	Tislelizumab Ociperlimab Pembrolizumab Placebo	Non-small cell lung cancer	Recruiting	Phase 3	June 8, 2021
NCT04736173	Zimberelimab Domvanalimab Carboplatin Pemetrexed Paclitaxel	Non-small cell lung cancer Nonsquamous non-small cell lung cancer Squamous non-small cell lung cancer Lung cancer	Recruiting	Phase 3	February 1, 2021
NCT04732494	Tislelizumab Ociperlimab Placebo	Esophageal squamous cell carcinoma	Recruiting	Phase 2	March 31, 2021
NCT04693234	Tislelizumab Ociperlimab	Cervical cancer	Active, not recruiting	Phase 2	March 3, 2021
NCT04672356	IBI939 Sintilimab	Advanced lung cancer	Recruiting	Phase 1	January 25, 2021
NCT04656535	AB122 AB154 Placebo	Glioblastoma	Recruiting	Early Phase 1	April 21, 2021

Epigenetic modifications more and more play a role in the upregulation of immune checkpoints in cancer. Through qRT-PCR, CpG methylation, and repressive histone abundance experiments, TIGIT was found poorly expressed in primary breast cancer and adjacent non-cancerous tissues because its CpG islands at the promoter level were mostly hypermethylated (80-70%), while CpG islands of PD-L1 and LAG3 promoter were demethylated at 100% and 80-90%, respectively (130). In another study, using large-scale transcriptome data analysis of aggressive breast cancers, TIGIT was found to be highly and specifically expressed in aggressive breast cancer, and its pro-tumor activities were linked to immune-related genes (151). An in-depth analysis by the same authors revealed that TIGIT expression was positively correlated with T cells, CD8^+^ T cells, cytotoxic T cells, NK cells, B cells, DCs, and macrophages, but negatively correlated with neutrophils, endothelial cells, and fibroblasts (151). Furthermore, TIGIT expression was positively correlated with inflammation and immune response-related genes (LCK, HCK, MHC-I, MHC-II, STAT1, and interferon) (151). Accordingly, TIGIT expression seems closely related to higher malignant pathological types of breast cancer and might be a potential biomarker of breast cancer progression.

The role of epigenetics in TIGIT expression and immunotherapeutic sensitivity was also uncovered in gastric cancer. Increased TIGIT expression in gastric cancer appears to be a favorable event (152). TIGIT expression correlates with an active immune landscape, survival and immunotherapeutic sensitivity, and favorable prognosis, according to a bioinformatics-guided analysis. Patients with high TIGIT expression respond better to immunotherapy than those with low TIGIT expression (152).

The role of TIGIT in cancer progression was updated in bladder cancer. The failure of the antitumor immune response in bladder cancer was attributed to a subset of TIGIT^+^ Treg cells overexpressing interleukin IL-32 using single-cell sequencing technology on tissue and experiments in a mouse model (153). In support of this, the same study found that anti-TIGIT monoclonal antibodies, when used alone, have a dual effect: they boost the antitumor activities of T cells while decreasing IL-32, which in turn inhibits bladder cancer metastasis (153). Furthermore, in muscle-invasive bladder cancer, the worst clinical outcomes were attributed to a suppressive TME characterized by Th2 cells, Tregs, mast cells, neutrophils, and exhausted TIGIT^+^CD8^+^ T cells with low tumoricidal capacity that benefited from anti-PD-L1 and anti-TIGIT immunotherapy (154, 155). However, in patients with stage II of muscle-invasive bladder cancer with low TIGIT^+^ CD8^+^ T cell infiltrate, adjuvant chemotherapy prolongs their OS and recurrence-free survival (RFS) (155). Therefore, TIGIT^+^ T cells have a prognostic role in clinical outcomes in bladder cancer and seem to be a predictive biomarker for inferior adjuvant chemotherapy responsiveness.

The CD155–TIGIT pathway suppresses the immune system at different levels in colorectal cancer. In colorectal cancer patients and mouse models, the TME is populated by exhausted TIGIT^+^CD8^+^ T cells with co-expression of other IRs and low levels of pro-inflammatory cytokines (IFN-γ, IL-2, TNF-α) (103, 156). Furthermore, high TIGIT expression was linked to advanced disease, early recurrence, and lower survival rates (156), and with advanced TNM stage and better disease-free survival (DFS) in colorectal cancer patients with mismatch repair deficiency (157). Another study discovered a higher TIGIT^+^CD3^+^ T cell subpopulation in the peripheral blood and cancer tissue of colorectal cancer patients than in healthy donors (121). TIGIT^+^CD3^+^T cells were exhausted cells with decreased proliferation, cytokine production, and glucose metabolism (121). TIGIT blockade, combined with PD-1 blockade, reversed these pro-tumorigenic features in the human MC38 colorectal xenograft mouse model. According to this data, GSEA computational analysis revealed that TIGIT expression in colorectal cancer drives the negative regulation of cytokine-cytokine receptor interaction pathway, chemokine signaling, and cytotoxic function of NK cells (122). *In vitro* studies have revealed that CD155–TIGIT pathway suppresses the downstream effector NF-κB, which is usually involved in the production of IFN-γ by NK cells, which in turn would activate cytotoxic CD8^+^ T cells (103). On the contrary, the same authors demonstrated that knocking out CD155 in colorectal cancer cells promotes the effector function of tumor-infiltrating CD8^+^ T cells, and inhibition of the CD155–TIGIT pathway suppresses the tumor growth in an *in vivo* mouse model. Overall, TIGIT^+^ cells in colorectal cancer were linked to advanced disease, early recurrence, and lower survival rates (103).

In pancreatic cancer, the CD155–TIGIT pathway suppresses immunity and promotes immune evasion (158, 159). The cancer progression of a subset of patients with pancreatic adenocarcinoma in metastatic/advanced stages was related to high-affinity MHC-I-restricted neoepitopes expression and exhausted TILs in the intratumoral compartment. Functional studies using orthogonal preclinical models revealed a synergistically antitumor response when TIGIT/PD-1 co-blockade was combined with CD40 agonism because they had been reinvigorated tumor-reactive T cells (158).

TIGIT^+^ immune cells were also shown to play a role in cancer invasion and metastasis in esophageal carcinoma. A transcriptomic profile investigation followed by immunohistochemistry validation has identified the allograft inflammatory factor 1 (AIF1) gene as an unfavorable prognostic factor in this carcinoma and demonstrated that it is associated with immune infiltrates (160). In the tumor infiltrate, T cells and NK cells are affected by AIF1, which promotes TIGIT expression, and hence induces or strengthens immunotherapy resistance sustained by an immune infiltrate enriched in Th1 cells and exhausted T cells.

According to mRNA profiling of CD8^+^ T cells in a murine model of autochthonous liver cancer, TIGIT is a hallmark of T cell exhaustion in liver cancer at various stages of their differentiation (161). TILs from patients with primary hepatocellular carcinoma and intrahepatic cholangiocarcinoma had an increased TIGIT^+^CD8^+^ T cell subpopulation. However, two subsets of these patients were identified: one had significantly higher TIGIT and PD-1 expression levels in the tumor area than the surrounding peritumoral area; whereas the other had a similar level of expression for both IRs in the tumoral and peritumoral areas (161).

In renal cell carcinoma (RCC), immunohistochemistry and flow cytometry experiments to evaluate TIGIT and PD-1 expression in circulating immune cells and TILs revealed an increased TIGIT and PD-1 expression in the tumoral area compared with adjacent normal tissue, but TIGIT^+^ T cells and NK cells amount did not correlate with clinicopathological characteristics (age, sex, tumor diameter, Fuhrman grade, or TNM stage) (162). In contrast, a positive correlation with RCC clinicopathological characteristics was observed only for PD-1 (162).

CD155 and TIGIT were correlated with clinicopathological features in lung adenocarcinoma, in which CD155 expression was strongly associated with tumor staging and poor OS (111). TIGIT expression was associated with advanced TNM staging, which correlated with lymphatic metastasis and distant metastasis, with low antitumor immunity-related gene expression activation and poor PFS (111).

In oral squamous cell carcinoma, circulating T cells and TILs overexpressed TIGIT on CD4^+^ and CD8^+^ T cells, characterized by dysfunctional phenotype, including reduced proliferative capacity and low proinflammatory cytokine release (163). Higher TIGIT expression was also associated with higher T stage and nodal invasion but not with other clinicopathological variables such as age, gender, smoke/alcohol use, tumor site, and tumor differentiation (163).

Singer et al. proposed TIGIT expression as a predictive rather than prognostic biomarker for reactive tumor-infiltrating immune cells in soft sarcoma tissue in an elegant investigation on IL-15 and TIGIT blockade therapy to reactive tumor-infiltrating immune cells (164). The authors observed both activated and exhausted tumor-infiltrating NK cells and TILs and TIGIT upregulation in the TME, especially on NK cells, associated with superior distant disease recurrence-free and OS (165). Interestingly, activator and inhibitor pathways are not mutually exclusive and are a recent field of interest in targeted therapy (164, 165).

Under hypoxic conditions, HIF-1α transcript factor activation stimulates the expression of various IRs, including TIGIT. TIGIT and HIF-1α activity suppression experiments, using a siRNA carrier system, have revealed a critical role of these molecules in tumor growth, apoptosis, and metastasis in colorectal and breast cancer (166). In colorectal cancer cell line CT26 and breast cancer cell line 4T1 and in their *in vivo* mouse models, TIGIT and HIF-1α down-regulation diminished the colony formation ability and afflicted cancer cells’ angiogenesis and proliferation activities, suggesting simultaneous blocking of TIGIT and HIF-1α as a potential new treatment strategy (166).

Considering all these results, it is possible to speculate that later than tumorigenesis, when the tumor already presents an immune infiltrate, immune cells, particularly T cells, upregulate TIGIT, promoting an immunosuppressive microenvironment that leads to metastasis and unfavorable prognosis. The studies with an in-depth microenvironment characterization and association with clinicopathological features point out several diverse IRs expression combined analysis that might represent an effective outcome prediction panel in cancer. However, there is much work to be done to understand in more detail TIGIT’s role in the different tumor stages (e.g., initial diagnosis, progression, recurrence, metastases) in various cancers.

## Therapeutic Strategies Targeting TIGIT Immune Checkpoint Expression

Cancer treatments are traditionally based on surgery, targeted therapies, chemotherapy, or radiation therapy (167). Immune inflammatory modulation-based therapy, or more simply immunotherapy, has lately emerged as a novel therapeutic arm with enormous potential, particularly in the treatment of cancer chemo-radiotherapy resistance (168, 169). Immunotherapy is a type of treatment that aids the immune system in fighting cancer and other diseases.

Immunotherapies have been shown to be effective against tumor-associated T cells that are dysfunctional. The rationale behind these therapies is that the cancer cells overexpress ligands for IRs, such as CD115, CD112, and others, to elude the immune system. Different immunotherapy strategies aim to boost the patient’s antitumor immune response against malignancies minimizing T cell exhaustion and providing protective effects against recurrence and metastasis with less toxicity when compared to traditional cancer therapy (170).

Here is an update on therapeutic strategies targeting TIGIT immune checkpoint expression.

Cancer immunotherapy strategies that boost innate and adaptive immunity are being developed to achieve long-lasting antitumor effects. Azelnidipine is a long-acting third-generation dihydropyridine calcium channel blocker that has been approved for the treatment of hypertension. However, using the molecular operating environment (MOE) by blocking and MST binding assays, molecular docking and structural analysis of CD172a and CD112 have indicated azelnidipine’s potential relevance in cancer immunotherapy (171). Azelnidipine inhibits the innate checkpoint CD47/CD172a and the adaptive checkpoint TIGIT–CD112 pathways and has anti-cancer effects by increasing the infiltration and function of CD8^+^ T cell and macrophage tumor cell phagocytosis *in vivo* and *in vitro*. This study extensively looked at the effect of TIGIT blockade on macrophages in the tumor. Tumor cells, like normal cells, can express CD47, a “do not eat me” signal that prevents CD172a^+^ macrophages from phagocytosing them. Zhou et al. demonstrated that azelnidipine blocks CD47–CD172a signaling, reactivates macrophage phagocytosis, and improves antitumor immunity even in combination with radiotherapy, as shown in the MC38 murine colon adenocarcinoma cell line (171). A cancer immunotherapy antibody targeting both CD47 and TIGIT has been patented (WO2020259535).

Alternative anticancer treatments with a systemic approach are being developed. In a mouse model of lung adenocarcinoma, triple therapy with the RadScopal approach (high-dose radiation to primary tumors plus low-dose radiation to secondary tumors) plus anti-TIGIT and plus anti-PD-1 prolong survival and block tumor growth while decreasing TIGIT^+^ exhausted T cells and TIGIT^+^ Tregs (104). This approach promotes a systemic antitumor response because low-dose radiation also reduces CD155 expression on TAMs and DCs (104). Combined therapies based on immunotherapy and radiation therapy promise to reset the TME.

TIGIT^+^ macrophages were also looked at in leukemia, in which the leukemia-associated macrophages (LAM) co-expressing TIGIT, TIM3, and LAG3 were identified as immunosuppressive M2 responsive to *in vitro* TIGIT blockade therapy that polarizes the M2 toward the M1 phenotype and improves phagocytosis of the CD47 expressing tumor cells (172, 173).

An in-depth characterization of TILs in bladder cancer using PBMC isolation and tumor single-cell isolation from fresh tumor tissue demonstrates that PD-1^high^TOX^+^ T cells play a key role in tumor evasion, which might be reversed by combining PD-1 and TIGIT inhibition (174).

pt?>Autophagy, a cell-intrinsic system that uses the lysosome to remove damaged organelles and proteins, plays a critical role in cellular immunity. Indeed, autophagic abnormalities linked with oncogenesis promote tumor escape by influencing cell immunogenicity, APC activity, and T cell activity (175). Artesunate, an anti-malaria drug, exerts anticancer activity by inhibiting proliferation, migration, and angiogenesis and inducing apoptosis and autophagy. Artesunate-induced autophagy was well demonstrated in human bladder cancer cells, upregulating ROS and activating the AMPK-mTOR-ULK1 axis and in uterine corpus endometrial carcinoma, enhancing NK cell cytotoxicity *via* interactions with tumor cells overexpressing CD155, and upregulating co-stimulator CD226 and downregulating co-inhibitor TIGIT (176–178).

Anti-TIGIT antibodies are used instead in more consolidated therapies. TIGIT blocked reduced tumor growth while promoting an immune infiltration enriched in effector cytokine-secreting CD8^+^ T cells (44, 116, 127, 179).

Vibostolimab is a humanized antibody that targets TIGIT preventing its binding with CD112 and CD155. Patients with advanced solid tumors who received vibostolimab alone or combined with the anti-PD-1 pembrolizumab in a phase I clinical trial (NCT02964013) showed controllable tolerance across escalating doses and all types of advanced solid tumors assessed. Increased NK cell activation of CD8^+^ T cells was found to have an anticancer effect in the study (180).

Etigilimab is another anti-TIGIT monoclonal antibody that is now being investigated in an open-label, multicenter, phase I/II clinical trial (NCT04761198) in patients with advanced or metastatic solid tumors for tolerance and pharmacokinetics with the anti-PD-1 nivolumab (181).

Combining anti-TIGIT and anti-PD-1 immunotherapy in metastatic melanoma has shown encouraging outcomes, with increased proliferation, cytokine generation, and degranulation of effector CD8^+^ T cells (116).

Mono- or dual TIGIT and PD-1–PD-L1 blockade aims to take advantage of the curative potential of pre-existing tumor-primed T cells in cancer treatment by promoting CD8^+^ T cell proliferation and function, resulting in protective memory T cells that ensure tumor rejection and avoid recurrence (182–184). Although several of these antibodies have received clinical approval, their effectiveness remains modest because immunological checkpoints and their signaling are regulated at multiple levels.

In addition to monoclonal antibodies, the most recent approach is to design T cells for TIGIT.

Hoogi et al. created a TIGIT : CD28 chimeric co-stimulatory switch receptor with the TIGIT exodomain fused to the CD28 signaling domain, which improved the activities of chimeric antigen receptor T cells by stimulating cytokine production and activating other T cell effector functions (185).

## Efficacy And Toxicity of Anti-TIGIT Immune Checkpoint Therapy

Even though therapeutic strategies targeting immunological checkpoints have been approved for a variety of cancer types, patients continue to have poor prognoses and suffer from immune-related adverse events (irAEs) that affect numerous organs. irAEs are secondary to the infiltration of activated T cells and can affect any organ (186, 187). Skin, gastrointestinal tract, endocrine, lungs, thyroid, pituitary and adrenal glands, and the musculoskeletal system are the most usually impacted, while nervous, renal, hematologic, ophthalmic, and cardiovascular systems are less commonly affected (188–190). Four degrees of irAEs can be distinguished based on the organs involved and the severity: patients with grade 1 irAEs show skin toxicity (<10% body surface area) and no sign of toxicity for the gastrointestinal tract, liver, endocrine system, and lungs; patients with grade 4 of irAEs show elevated skin toxicity (> 30% body surface), hepatotoxicity, and severe symptoms of involvement of the cardiovascular, endocrine, and digestive apparatus (191). Grade 2 and 3 show intermediate signs. The management of irAEs is based on well-established clinical practice guidelines well reviewed by Barber in 2019 (192). Some irAEs are more common in immune therapy than chemotherapy, and their frequencies are positively associated with clinical efficacy, making them useful for clinical decisions (193).

To understand the efficacy and toxicity of immune checkpoint therapy, it should be noted that the types of antibodies used in anti-TIGIT therapies are very different and more or less tolerated. A murine, chimeric, humanized, or completely human IgG antibody could be used to suppress immunological checkpoints (194). The majority of anti-TIGIT antibodies in clinical trials are either humanized (such as ociperlimab, pembrolizumab, atezolizumab) or fully human (such as tiragolumab, etiligimab, ipilimumab, nivolumab, vibostolimab, domvanalimab) (195). Compared to other forms of IgG origin, humanized and completely human antibodies have increased *in vivo* tolerability but much-reduced immunogenicity (194).

Furthermore, blockade therapy efficacy depends on the antibody-dependent cellular cytotoxicity (ADCC) desired to destroy unfunctional T cells and tumor cells. ADCC is a non-phagocytic mechanism in which antibody-bound target cells are killed by innate immune cells such as NK cells, DCs, and macrophages (196). To activate ADCC, the targeted cell must express target antigens, the antibody must be preferentially IgG1 or IgG3 monoclonal because these two antibodies link any type of FcR, and the effector cell must have the Fc-gamma receptors (FcγR) (196). Concerning TIGIT, its FcγR is active in tiragolumab, ociperlimab, vibostolimab, EOS-448, etigilimab, and AGEN-1307, whereas it is inactive in domvanalimab, BMS-986207, and CASC-674 (195). However, FcγR presence or absence has not been tested for anti-TIGIT antibody clinical efficacy (197, 198).

Recently, TIGIT molecular was also used as Fc-fused protein in some reports demonstrating that TIGIT-Fc may act both as an immunosuppressor and as an immunostimulator in a microenvironment-dependent way (83, 85, 199–201). TIGIT-Fc is a dimer in which an Fc domain of an antibody is linked to the extracellular domain of TIGIT by covalent bonds. TIGIT-Fc has antibody-like features, such as a long serum half-life and efficient expression and purification *in vitro*, making it an ideal drug (46). Its action as an immunosuppressor was demonstrated *in vitro* and in a mouse model of acute allogeneic GVHD in which it decreased CD8^+^IFN-γ^+^ and CD8^+^ granzyme B^+^ T cells activation in a dendritic cell-dependent manner and reduced the release of IL-10 (83).

Moreover, TIGIT-Fc acts as a negative regulator of inflammation, inhibiting macrophage activation and imbalanced M1/M2 ratio in favor of M2 anti-inflammatory profile *via* c-Maf up-regulation, which promotes IL-10 transcription as demonstrated by *in vivo* and *in vitro* experiments using fibroblasts stably secreting TIGIT-Fc in the LPS shock model (85). In CLL, a tumor-supportive role of TIGIT^+^CD4^+^ T cells was observed in the presence of TIGIT-Fc *via* down-regulation of IFNγ and IL-10 production (201). Interestingly, this protumor activity of CD4^+^ T cells was dependent on CLL cell’s presence because *in vitro* experiments with CD4^+^ T cells alone did not show any effects (201).

On the contrary, TIGIT/ligand interactions using recombinant TIGIT-Fc molecule immunostimulatory functions were shown in xenograft mouse models containing different human tumor cells (A375, A431, SK-BR-3, SK-OV-3, and H2126) co-implanted with human T cells (200). The TIGIT-Fc treatment enhanced effector NK cell functions and activated an anti-tumor T cell immune response *via* CD4^+^ T cells preventing their exhaustion (200). Additionally, synergistic effects were observed in TIGIT-Fc plus anti-PD-L1 combined therapy (200).

Efficacy and toxicity of anti-TIGIT therapy were evaluated in the CITYSCAPE trial (NCT03563716), in which anti-TIGIT (tiragolumab) with anti-PD-L1 (atezolizumab) combined therapy were applied. The findings showed that this combined therapy in non-small cell lung cancer (NSCLC) is well tolerated when compared to CTLA4 with PD-L1 combined therapy, and that it improves responses and PFS in PD-L1–immune sensitive patients (202–205). Furthermore, despite the similar safety profiles of atezolizumab with placebo (AP) vs. atelozomab with tiragolumab (AT), 80.6% of patients in the AT group and 72% of patients in the AP group suffered irAEs. The irAEs included rash and thyroid issues, infusion reactions at the first dose, soft stool, diarrhea, and very few cases of more severe toxicities, like hepatitis (204, 205).

The anti-TIGIT vibostolimab was tested in patients with solid tumors as monotherapy or in combination with the anti-PD-L1 pembrolizumab in the phase I multicohort MK-7684-001 trial (NCT02964013). The ORR for vibostolimab monotherapy was more significant than for combination therapy in the sub-cohort of NSCLC patients with anti–PD-1–PD-L1–refractory disease (7% (95% CI, 2%-20%) vs. 5% (95% CI, <1%-18%)) (206). IrAEs were reported by 65% of patients in the same NSCLC sub-cohort, including pruritus, fatigue, rash, arthralgia, decreased appetite, and 13% also had lipase elevation and hypertension (206).

TIGIT blockade therapy may be more beneficial if it is evaluated as a first-line treatment. In February 2020, a multicenter, open-label, phase I/II study using the novel anti-TIGIT EOS884448 as monotherapy was launched in patients with previously treated advanced cancer (ovarian, head and neck, cervical, and colorectal) (NCT04335253) (207). Multiple mechanisms of action for EOS884448 were demonstrated: inhibition of TIGIT triggering activation of TIGIT^low^ T cells and NK cells; depletion of immunosuppressive Treg and exhausted TIGIT^high^ T cells; and reverse activation *via* FcγR engagement (207). The pharmacokinetic and pharmacodynamic analysis demonstrated that exhausted Tregs and TIGIT^+^ T cells were depleted in a dose-dependent manner. Moreover, in this interventional study with multiple ascending-dose treatments, EOS884448 was generally well tolerated at all tested doses in patients with advanced cancer and had a promising antitumor activity as a single agent also in PD1-resistant patients. IrAEs were reported by 82% of patients, including pruritus, infusion-related reaction, fatigue pyrexia, rash macuolo-papular, eczema, and hypothyroidism (207).

Cancer patients’ stratification based on tumor response to immune checkpoint inhibitors is vital even if challenging to evaluate (208, 209). In fact, patients might experience clinical pseudoprogression that can be misinterpreted as disease progression because it cannot be evaluated with the existing response-evaluation criteria (210, 211). In tumor pseudoprogression, an increase in tumor size depends on infiltrating T cells, while in proper tumor progression, the increased tumor mass is due to proliferating tumor cells (210). In 2017, the Response Evaluation Criteria in Solid Tumors (RECIST) working group published a modified set of response criteria, the immune-related response criteria (iRECIST), adapted for immunotherapy because of the importance of a standardized strategy to evaluate its effects (212, 213).

Identifying prognostic biomarkers of response to TIGIT blockade alone or in combination with other IRs is needed to improve efficacy and reduce toxicity.

## Challenges and Conclusions

To summarize, in cancer, the genetic and epigenetic alterations could initiate tumorigenesis, which activates T cells and NK cells, and TME gets infiltrated by immune cells. Following T cells and NK cells upregulate TIGIT expression, which leads to an immunosuppressive TME, promoting tumor progression, immune escape, and metastases that result in poor prognosis.

Immune inflammatory modulation-based therapy is a promising therapeutic strategy against solid and hematological malignancies, but the outcomes are not largely encouraging because some tumor types remain refractory primarily to these therapies (214). CD8^+^ T cells are extremely heterogeneous, while CD4^+^ T cells in immunosuppression and immunotherapy are under-investigated (44). Targeting only a part of the complicated tumor system is insufficient for most cancer therapies or only in the arm of immunotherapies, so patients cannot benefit for a long time. New combined multiple targets (other co-inhibitory receptors) for immunotherapy must be explored to improve treatment.

Guidelines should be set for immunotherapy research. The results of different studies are difficult to compare due to the different designs for types of cancer, sample size, and statistical analysis. Consequently, when the results of individual studies are analyzed, they are insufficient to adopt particular and successful therapeutic interventions.

Side effects of traditional and immune checkpoint blockade therapies should be evaluated in-depth. High cytokine release and effector cell infiltration into TME cause irAEs that sometimes lead to the death of patients (215–217). Skin, gastrointestinal tract, lung, or liver are all affected by irAEs. However, the TIGIT blockade seems to have fewer side effects compared with other IRs blockades, as demonstrated in TIGIT^-/-^ mouse model (218–220). In this pre-clinical model, TIGIT blockade triggers fewer irAEs than anti-PD1 or anti -CTLA4 therapies (218–220).

Anti-TIGIT therapy is now being tested in 25 clinical trials, considering only those starting from 2021 (**Table 1**), but there is still considerable work to be done to discover new and safely targetable immune checkpoints that could be effective against various malignancies.

The immunological and stromal characterization of the TME cells and their amount and spatial distribution in relation to pathology and prognosis will help patient stratification, enhance personalized cancer therapy efficiency, and overcome tumor immune evasion mechanisms.

## Author Contributions

This work was conceived and planned by TA. The original draft preparation and writing: TA. Review and editing: DR and RT. All authors have read and agreed to the published version of the manuscript.

## Funding

This work was supported by Associazione “Il Sorriso di Antonio”, Corato, Italy and by ARES-Centro Salute Ambiente Progetto Ionico-Salentino.

## Conflict of Interest

The authors declare that the research was conducted in the absence of any commercial or financial relationships that could be construed as a potential conflict of interest.

## Publisher’s Note

All claims expressed in this article are solely those of the authors and do not necessarily represent those of their affiliated organizations, or those of the publisher, the editors and the reviewers. Any product that may be evaluated in this article, or claim that may be made by its manufacturer, is not guaranteed or endorsed by the publisher.

## Glossary

**Table:**

ADCC	antibody-dependent cellular cytotoxicity
AIF1	allograft inflammatory factor 1
CIK	cytokine-induced killer
CLL	chronic lymphocytic leukemia
CTLA4	cytotoxic T-Lymphocyte Antigen 4
DFS	disease-free survival
DCs	dendritic cells
DNMTs	DNA methyltransferases
FcγR	Fc-gamma receptors
GSEA	Gene set enrichment analysis
HiNeo	highly expressed neoantigens
IRs	inhibitor receptors
irAEs	immune-related adverse events
IRF4	IFN regulatory factor 4
ITIM	immunoreceptor tyrosine-based inhibitor motif
ITT	immunoglobulin tyrosine tail
LAM	leukemia-associated macrophages
MALs	malignant ascites lymphocytes
MHC-I	major histocompatibility complex class I
MMR	mismatch repair
MSI	microsatellite instability
NECL	nectin and nectin-like
NK	natural killer
NSCLC	non-small cell lung cancer
OS	overall survival
PBLs	peripheral blood lymphocytes
PD-1	programmed cell death protein 1
PD-L1	programmed cell death protein 1 ligand
PFS	progression-free survival
PVR	Poliovirus receptor
RCC	renal cell carcinoma
RFS	recurrence-free survival
RMST	restricted mean survival time
RORc	retinoic acid-related orphan receptor c
SHIP-1	SH2-containing inositol phosphatase-1
TAMs	tumor-associated macrophages
TANs	tumor-associated neutrophils
TCGA	The Cancer Genome Atlas
TIGIT	T-cell immunoreceptor with immunoglobulin and ITIM domain
TILs	tumor-infiltrating lymphocytes
TMB	tumor mutation burden
TME	tumor microenvironment
TNF	tumor necrosis factor
Tregs	regulatory T-cells
UCEC	uterine corpus endometrial carcinoma
VSIG9	V-set and immunoglobulin domain-containing protein 9
VSTM3	V-set and transmembrane domain-containing protein 3
